# Diffusion Behavior of Polyurethane Slurry for Simultaneous Enhancement of Reservoir Strength and Permeability Through Splitting Grouting Technology

**DOI:** 10.3390/polym17182513

**Published:** 2025-09-17

**Authors:** Xiangzeng Wang, Fengsan Zhang, Jinqiao Wu, Siqi Qiang, Bing Li, Guobiao Zhang

**Affiliations:** 1 Yanchang Petroleum (Group) Co., Ltd., Xi’an 710061, China; sxycpcwxz@126.com; 2 Natural Gas Research Institute of Yanchang Petroleum (Group) Co., Ltd., Xi’an 710061, China; jinqiaowu@163.com; 3 School of Engineering and Technology, China University of Geosciences, Beijing 100083, China; qiangsiqi@email.cugb.edu.cn (S.Q.); bing@cugb.edu.cn (B.L.)

**Keywords:** polyurethane slurry, dual-enhanced stimulation, finite-discrete element, fracturing pressure, coalbed methane

## Abstract

A polyurethane slurry was developed to simultaneously enhance the strength and permeability of geological formations, differing from the conventional fracture grouting used for soft-soil reinforcement. Injected via splitting grouting, the slurry cures to form high-strength, highly permeable channels that increase reservoir permeability while improving mechanical stability (dual-enhanced stimulation). To quantify its diffusion behavior and guide field application, we built a splitting-grouting model using the finite–discrete element method (FDEM), parameterized with the reservoir properties of coalbed methane (CBM) formations in the Ordos Basin and the slurry’s measured rheology and filtration characteristics. Considering the stratified structures within coal rock formed by geological deposition, this study utilizes Python code interacting with Abaqus to divide the coal seam into coal rock and natural bedding. We analyzed the effects of engineering parameters, geological factors, and bedding characteristics on slurry–vein propagation patterns, the stimulation extent, and fracturing pressure. The findings reveal that increasing the grouting rate from 1.2 to 3.6 m^3^/min enlarges the stimulated volume and the maximum fracture width and raises the fracturing pressure from 26.28 to 31.44 MPa. A lower slurry viscosity of 100 mPa·s promotes the propagation of slurry veins, making it easier to develop multiple veins. The bedding-to-coal rock strength ratio controls crossing versus layer-parallel growth: at 0.3, veins more readily penetrate bedding planes, whereas at 0.1 they preferentially spread along them. Raising the lateral pressure coefficient from 0.6 to 0.8 increases the likelihood of the slurry expanding along the beddings. Natural bedding structures guide directional flow; a higher bedding density (225 lines per 10,000 m^3^) yields greater directional deflection and a more intricate fracture network. As the angle of bedding increases from 10° to 60°, the slurry veins are more susceptible to directional changes. Throughout the grouting process, the slurry veins can undergo varying degrees of directional alteration. Under the studied conditions, both fracturing and compaction grouting modes are present, with fracturing grouting dominating in the initial stages, while compaction grouting becomes more prominent later on. These results provide quantitative guidance for designing dual-enhanced stimulation to jointly improve permeability and mechanical stability.

## 1. Introduction

Coalbed methane (CBM) is a hydrocarbon gas primarily composed of methane, which is typically adsorbed onto coal matrix particles or exists as free gas in the pores or water within coal seams. CBM poses a safety risk to underground coal mining operations [1,2]. Initially, CBM was extracted prior to coal mining, but with advancements in coalbed drilling and completion technologies, as well as increasing energy demand, CBM has evolved into a commercially viable clean energy source. The global proven reserves of CBM are approximately 256.3 × 10^12^ m^3^, which accounts for about 50% of the proven reserves of conventional natural gas [3]. Countries like the United States and the United Kingdom were pioneers in CBM development, and now have large-scale commercial CBM industries [1,4,5]. In recent years, deep CBM has become a major focus of research due to its increasing exploration and development. Deep CBM refers to CBM resources in coal seams where vertical geostress exceeds horizontal geostress, typically under conditions of high geostress, geothermal temperature, and reservoir pressure [6,7]. China has vast deep CBM resources, with proven reserves reaching 40.97 trillion m^3^ [8,9]. However, the geological conditions in deep CBM [10,11,12], such as high geostress and geothermal temperature, result in coal reservoirs that exhibit lower porosity and permeability, often dominated by clogged pores [7,13]. Studies show that increased temperature and pressure negatively affect CBM adsorption, reducing the reservoir’s adsorption capacity [14]. Therefore, proper investigation is required to resolve problems for the large-scale, efficient development of deep CBM.

The original structure of deep coal seams is complex, featuring low permeability, low gas saturation, and natural bedding development [15]. To enhance the production efficiency of deep CBM, hydraulic fracturing technology is primarily utilized. Hydraulic fracturing is a stimulation technology employed in the development of unconventional oil and gas fields, including shale gas and CBM [16,17]. In China, CBM fracturing operations are mainly concentrated in regions like the Junggar Basin and the Qingshui Basin. In the Junggar Basin, the average burial depth of coal beds in the Badawan Formation exceeds 2500 m. Large-scale fracturing was performed using a method that involved low-viscosity fluids, high displacement, substantial fluid volumes, and a high sand ratio, successfully fracturing coal beds at depths of up to 2567 m. This operation resulted in spontaneous blowouts, with subsequent stable gas production reaching approximately 7300 m^3^ per day [18,19]. Conversely, in the Qinshui Basin, the coalbed methane reservoir is characterized by deep, low-permeability formations, which lead to significant drilling depths and low average daily production rates. By employing low-concentration guar gum as the conventional fracturing fluid, followed by secondary unblocking fracturing with activated water, the daily gas production from a single well can exceed 2000 m^3^. However, field operations frequently encounter challenges such as sand plugging, which hampers the ability to achieve the desired sand addition and limits the effectiveness of production enhancements [20,21,22]. Many researchers have conducted studies to investigate the initiation and expansion mechanisms of hydraulic fracturing fractures. Li et al. [23] developed a calculation model for the initiation pressure of coal rock fractures based on an equivalent mechanical model of coal rock. Similarly, Jiang et al. [24] conducted hydraulic fracturing experiments on large coal samples, which revealed the mechanisms behind the formation of fracture networks; Zangeneh et al. [25] utilized a two-dimensional discrete element method to simulate the initiation and propagation of hydraulic fractures. Furthermore, coal reservoirs often contain numerous natural bedding planes, which can intersect with hydraulic fractures following the hydraulic fracturing process, altering their direction [26]. Ma et al. [27] suggested that the formation of fracture networks is more likely when main fractures and natural fractures intersect, resulting in increased CBM production. Liu et al. [28] indicated that natural fractures tend to redirect hydraulic fractures in the direction of the maximum horizontal principal stress. Additionally, various engineering factors can influence the interaction between hydraulic and natural fractures; injection rates below 15 bbl/min—and fluid viscosity near 1 cP—tend to steer hydraulic fractures along natural fractures, whereas rates around 60 bbl/min combined with viscosities near 100 cP favor penetration [29]. However, most studies have focused on shallow coal reservoirs, and there has been limited exploration of the propagation characteristics of hydraulic fractures in deep CBM reservoirs, as well as the differences in hydraulic fracture expansion between deep and shallow coal reservoirs. Therefore, further research into the propagation dynamics of hydraulic fractures in deep CBM reservoirs is essential.

In addition to conventional hydraulic fracturing, Li et al. [30] introduced the concept of splitting and grouting to stimulate reservoirs. This method involves high-pressure pumping of a specially formulated slurry that splits within the reservoir to create porous veins, thereby enhancing permeability. Recently, there has been increasing interest in splitting grouting technology, leading to continuous improvements in slurry properties. One widely used material is phenolic foam grouting, known as Roquefort Foam, which solidifies into a highly porous and strong cemented body just minutes after injection, expanding up to 15 times its original volume [31]. Additionally, Sun et al. [32] developed a dual-increasing stimulation slurry based on polyurethane for hydrate reservoirs in the South China Sea. This slurry quickly cures to form a highly permeable and strong framework while effectively bonding with the surrounding formation, enhancing reservoir stability and permeability. Wang et al. [33] and Yu et al. [34] determined that the minimum principal stress is the primary factor influencing slurry vein propagation during splitting grouting, with the veins consistently propagating vertically along this stress. Additionally, reservoir non-homogeneity affects the morphology of the slurry veins.

**Figure 1:**
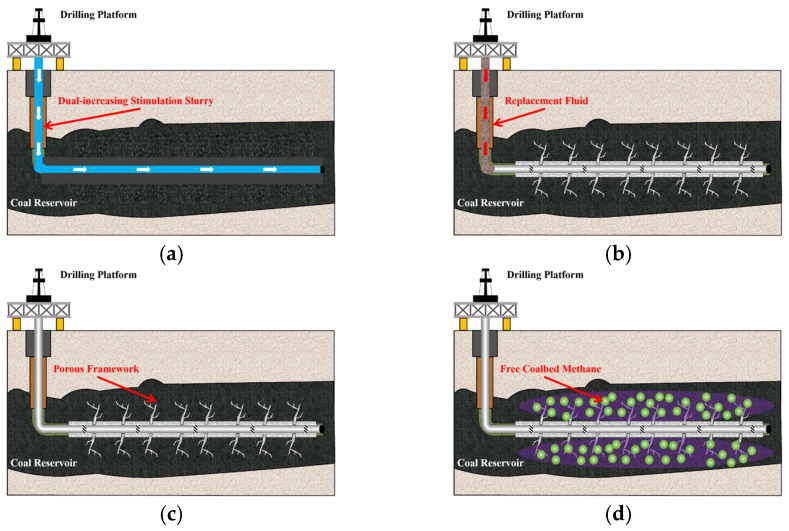
Dual-enhanced stimulation process of horizontal wells for CBM. (**a**) Injection of slurry for fracturing stimulation. (**b**) Injection of displacing fluid to replace the slurry. (**c**) Slurry solidification to form porous slurry veins. (**d**) Depressurization for CBM extraction.

Compared to conventional hydraulic fracturing, dual-enhanced stimulation technology offers superior performance in weak reservoirs by utilizing a specialized polyurethane slurry injected under high pressure. This slurry cures in situ to form high-strength, highly permeable channels that simultaneously enhance reservoir permeability and improve mechanical stability. The technology effectively addresses common issues such as sand blockage, proppant embedment, and flowback by creating a self-supporting fluid system that eliminates the need for external proppants. Additionally, it significantly expands the effective propping range of fractures and mitigates fine particle migration and blockage, thereby optimizing long-term conductivity and reservoir productivity. Based on this, the study proposes applying this technique for CBM extraction, as illustrated in Figure 1. First, the slurry is injected directly into the CBM reservoirs for splitting. A high-pressure pump is used to inject the slurry into the CBM reservoirs at a pressure exceeding the formation’s fracturing pressure, creating a slurry vein network. The pumping is stopped once the desired stimulation effect and threshold limit of slurry volume are achieved. Next, displacement fluid is injected into the wellbore to ensure that all dual-enhanced stimulation slurry reaches the reservoir, after which the pump is turned off and the well is closed. Once the dual-enhanced stimulation slurry has fully cured, it gradually forms a porous slurry vein network in the reservoir, enhancing both stability and permeability. Finally, pressure is reduced for extraction, allowing gas to be rapidly produced through the porous slurry veins. To understand the behavior of porous slurry vein propagation in CBM, a model for dual-enhanced stimulation splitting grouting in deep CBM reservoirs with natural bedding was developed using the finite-discrete element method, based on the characteristics of deep CBM reservoirs in the Ordos Basin of China. This study analyzes the effects of engineering parameters, geological factors, and bedding features on the propagation behavior of the slurry veins, focusing on the fracturing and diffusion processes during injection to create high-performance slurry veins. The study provides a theoretical foundation for determining process parameters for the dual-enhanced stimulation of CBM reservoirs.

## 2. Modeling and Parameterization

### 2.1. Finite-Discrete Element Methods

The finite-discrete element method (FDEM), proposed by Prof. Munjiza in 1995s [35,36], combines the strengths of finite element and discrete element methods to model dynamic motion and crack propagation in deformable materials [37]. In FDEM, the computational domain is divided into finite and discrete elements, with stress–strain solutions handled in the finite element domain and hydraulic crack extension in the discrete domain [38]. Crack propagation involves inserting cohesive units between rock blocks, where cohesive force varies with crack width, affecting fluid pressure and rock deformation [39]. This approach enables detailed analysis of fracture dynamics and associated parameters.

FDEM is commonly employed to simulate fracture initiation and propagation in coal reservoir stimulation [40]. In this model, the matrix unit represents the mechanical and seepage characteristics of the porous medium, allowing for precise calculations of pore pressure and effective stress. The cohesive unit models fluid flow within the unit and accounts for filtration loss along its interior. The model is capable of generating coal reservoirs with natural fractures and bedding through pre-processing. By dividing the coal seam network and integrating cohesive units with varying strengths and fracture energies, the simulation successfully differentiates between coal rock and natural beddings. The matrix unit embodies the coal reservoir, while the cohesive unit governs the deformation of the matrix to capture displacement and stress. When the traction separation displacement of the cohesive unit exceeds a specified threshold, it undergoes a sequence of events—loading, yielding, and plastic damage—effectively simulating the fracturing process of coal rock.

### 2.2. Parameterization

The simulated coalbed methane reservoir is located in the southeastern part of the Yishan slope in the Ordos Basin of central and western China, with the primary gas-bearing formations being the Shanxi Formation and the Benxi Formation. In the studied section, the coal seam is overlain by about 10 m of limestone (roof) and underlain by about 12 m of mudstone (floor). The seams are buried at depths greater than 2000 m, and the coal rock exhibits developed natural beddings. The coal rock contains a diverse range of minerals, with a high content of clay minerals and an average porosity of 5.5%. The CBM reservoir pore pressure is approximately 28.9 MPa, and there is a limestone layer about 10 m thick capping the coal seam, with vertical stress reaching up to 40 MPa. The horizontal stress ranges between 19.1 and 39.4 MPa, with a lateral stress coefficient of less than 1. Under high-pressure water jet conditions, fractures are prone to extend vertically along the reservoir direction, forming vertical hydraulic fractures [41,42,43].

**Figure 2:**
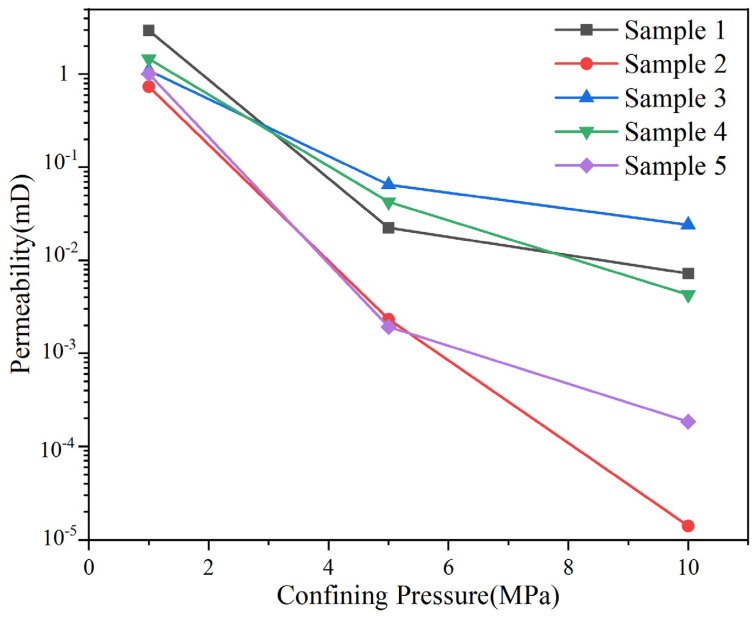
Permeability of coal cores under different confining pressures.

To obtain more accurate coal rock properties, the permeability and strength of coal rock samples were tested. Figure 2 illustrates the gas permeability of coal rock samples at various confining pressures. At a confining pressure of 1 MPa, the permeability averaged only 1.27 mD and dropped abruptly to below 0.1 mD with increasing confining pressure, mainly due to reduced fracture space and porosity, potentially decreasing by up to three orders of magnitude at a confining pressure of 10 MPa. This behavior is consistent with findings by Wu et al. [44] and Ya et al. [45], indicating significant stress sensitivity in deep coal rock permeability.

**Figure 3:**
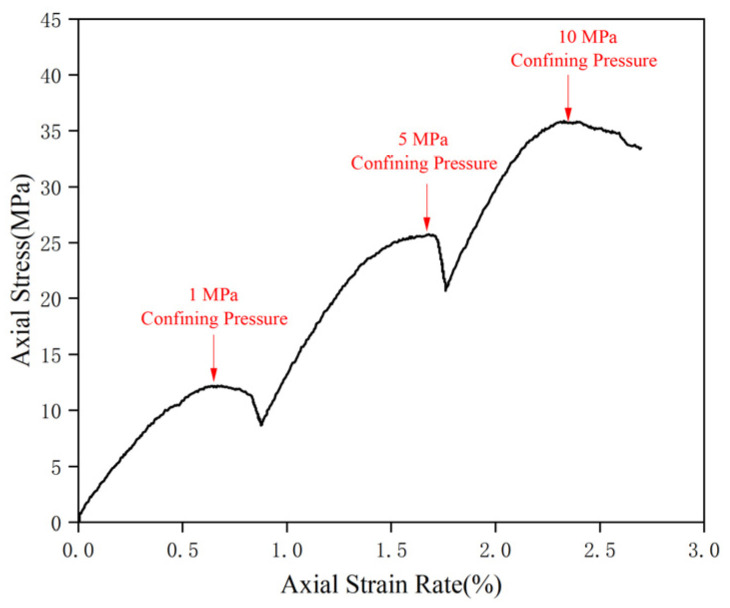
Triaxial stress–strain curve of coal cores.

**Figure 4:**
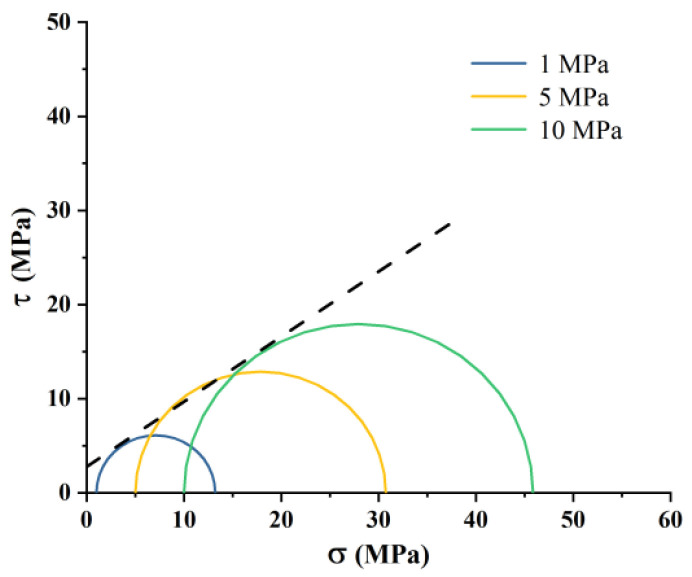
Mohr stress circle of coal cores.

Figure 3 illustrates the stress–strain curves of coal rock under different confining pressures, showing elastic-plastic deformation and clear strain-weakening characteristics. As confining pressure increases, the peak pressure rises from 12.18 MPa at 1 MPa to 35.81 MPa at 10 MPa, indicating the high mechanical strength of coal rock. To assess shear strength, the maximum compressive strength was plotted against surrounding pressure using the Moore-Cullen criterion, resulting in a cohesion of 4.64 MPa and an internal friction angle of 33.7° (Figure 4), which indicate that while the coal rock possesses some shear strength, it is still lower than that of the top and bottom tuffs.

The dual-enhanced slurry used in this study is a brownish-yellow, polyphase polyurethane slurry with a density of 1.68 g/cm^3^ and a viscosity that can be adjusted between 50 and 500 mPa·s. The composition and physical parameters of the dual-enhanced slurry can be found in the literature [32,46]. In this study, we primarily considered slurry density, viscosity, and filtration coefficient, referring to Table 1.

### 2.3. Dual-Enhanced Stimulation Model for Deep CBM Reservoirs

**Figure 5:**
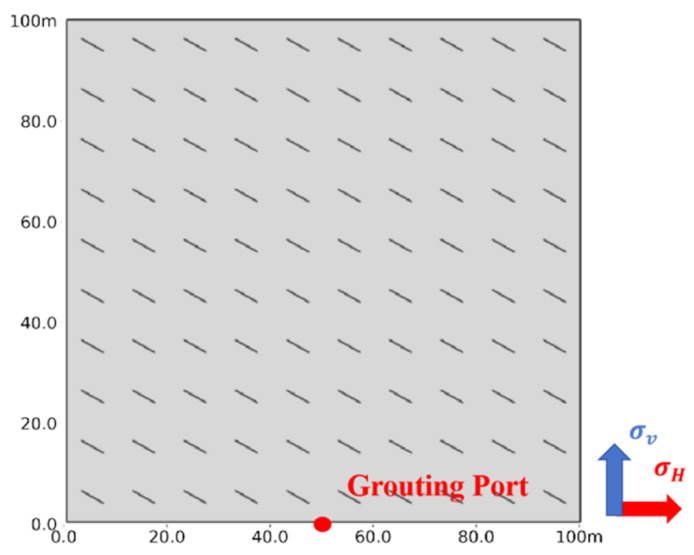
Split grouting model and mesh schematic.

**Figure 6:**
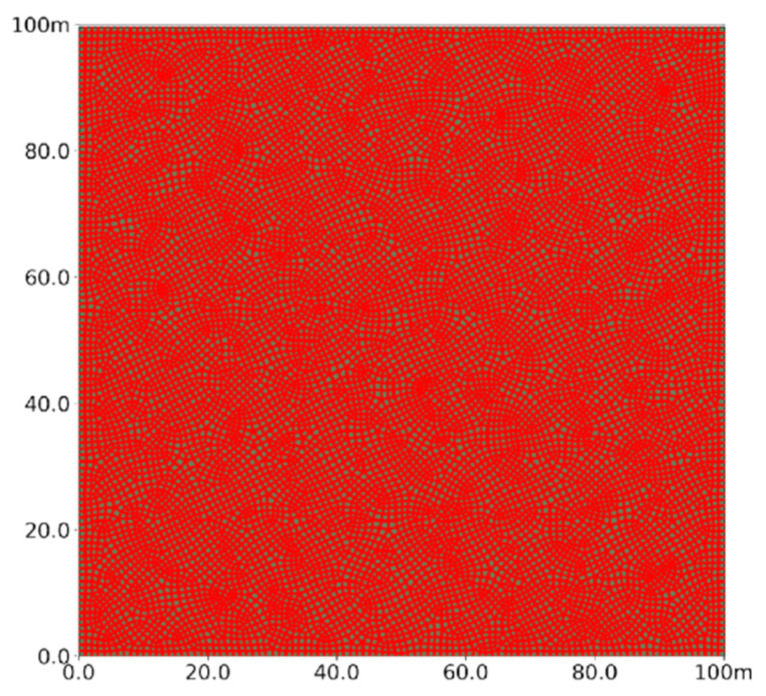
Model mesh schematic.

This study establishes a two-dimensional model using field data to analyze the vertical expansion of fractures in a horizontal well. The model simulates a 100 × 100 m deep coal seam, with its upper boundary buried at 1900 m. It includes the upper boundary, lateral boundaries, the grouting point, and the target coal seam, as shown in Figure 5. The coal seam is divided into coal rock and natural beddings using Python 3.7 code that interacts with the Abaqus CAE. The injection point is positioned at the bottom center of the model to inject dual-enhanced slurry into the CBM reservoir while restricting boundary displacement and pore pressure in other directions. Given the finite-discrete element method’s greater network dependence compared to traditional finite element methods [47], a finer mesh of 1 m is used, resulting in 17,795 CPE4P cells to accurately characterize seepage. Additionally, cohesive cells are inserted between neighboring CPE4P cells through secondary development, yielding a final model with 35,590 COH2D4P cell grids, as illustrated in Figure 6, with cohesive cells near the injection point designated as the initial damage type.

This paper investigates the initiation and extension patterns of fractures under various engineering parameters, geological factors, and natural beddings. The engineering parameters include a grouting rate of 1.2–3.6 m^3^/min, based on previous reservoir stimulation studies [48,49], and slurry viscosity, determined by the controllable characteristics of the dual-enhanced slurry. Geological factors involve the ratio of lateral pressure coefficient to the strength of natural beddings and coal rocks. Peng et al. [42] noted that the deep CBM reservoir in the study area typically exhibits vertical stress greater than horizontal stress, with lateral pressure coefficients ranging from 0.7 to 0.9. The strength of natural strata, influenced by their structure, length, and strike, usually does not exceed 40% of the coal rock matrix strength [50], and in this study, the strata strength to coal rock strength ratio is set at 10–30%. To explore the impact of natural bedding characteristics on fracture extension, six groups of bedding parameters were established, with inclination angles of 10°, 30°, and 60°, and surface densities of 25, 81, and 225 beddings per 10,000 m. More detailed parameters can be found in Table 2.

## 3. Results and Discussion

### 3.1. Influence of Engineering Parameters on Diffusion Behavior of Polyurethane Slurry

**Figure 7:**
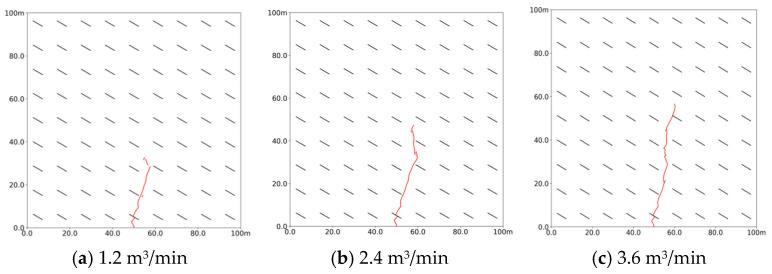
Propagation morphology of porous slurry veins at different injection rates. (Red lines denote slurry veins) (**a**) 1.2 m^3^/min. (**b**) 2.4 m^3^/min. (**c**) 3.6 m^3^/min.

As depicted in Figure 7, the slurry flows from the grouting point toward the natural bedding, following the direction of vertical geostress. After traveling along the bedding, the slurry turns and extends in the direction of the maximum principal stress, forming a curved slurry vein. Increasing the grouting rate significantly enhances both the propagation distance of the porous vein and the stimulated area of the reservoir. Notably, the expansion pattern of the slurry vein remains consistent across different grouting rates, with all instances showing a noticeable steering effect due to the presence of natural bedding.

**Figure 8:**
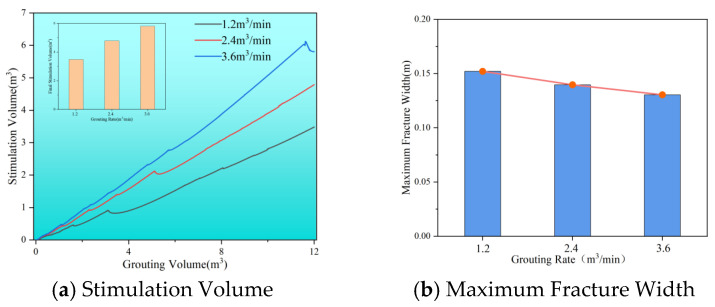
Stimulation volume and maximum fracture width at different injection rates. (**a**) Stimulation Volume. (**b**) Maximum Fracture Width.

Figure 8 illustrates that raising the grouting rate from 1.2 m^3^/min to 3.6 m^3^/min increases the stimulation volume from 3.48 m^3^ to 5.80 m^3^, while the maximum width of the slurry vein decreases from 0.15 m to 0.13 m. This indicates that a higher grouting rate directs more slurry along the main fracture, resulting in less residual slurry between the cracks and a narrower maximum crack width. It is essential to note that the stimulation volume of the slurry does not always increase with the grouting process; at certain rates, it may actually decrease. This can happen because the seepage of the slurry into the reservoir can further reduce fracture width, ultimately impacting the overall stimulation volume.

**Figure 9:**
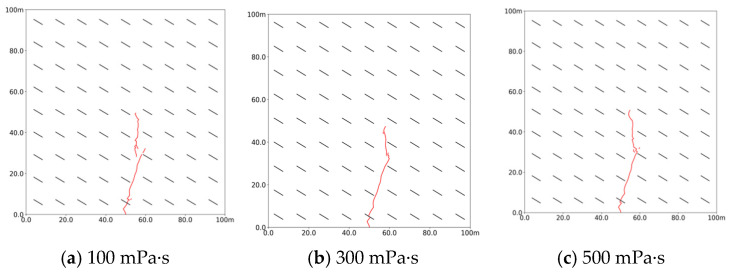
Propagation morphology of porous slurry veins with different slurry viscosities. (**a**) 100 mPa·s. (**b**) 300 mPa·s. (**c**) 500 mPa·s.

As shown in Figure 9, increasing the viscosity of the slurry from 100 mPa·s to 500 mPa·s significantly alters the morphology of the slurry veins. At higher viscosity, the slurry penetrates the bedding and extends along it, leading to a change in direction. During the forward propagation, the slurry veins transition from multi-branch to single-branch, although the maximum length of propagation remains nearly the same. This behavior indicates that lower viscosity allows the slurry to move more easily through the coal rocks, reducing resistance and enabling forward movement and splitting in the direction of maximum stress. Conversely, as viscosity increases, the filtration loss coefficient decreases, which results in slower diffusion rates and a reduced tendency to form multi-branched veins.

**Figure 10:**
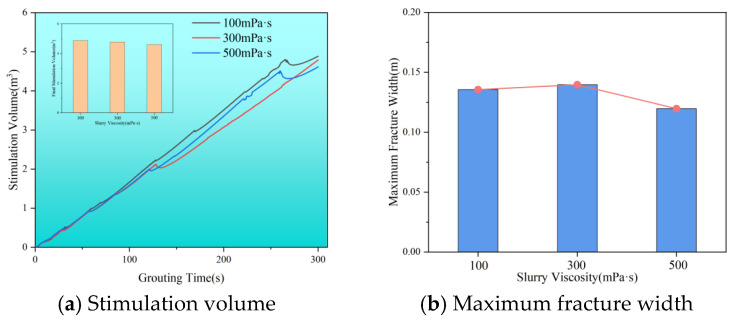
Stimulation volume and maximum fracture width at different slurry viscosities. (**a**) Stimulation volume. (**b**) Maximum fracture width.

As shown in Figure 10, the effective stimulation volume decreases as viscosity increases: 4.89 m^3^ at 100 mPa·s, 4.78 m^3^ at 300 mPa·s, and 4.61 m^3^ at 500 mPa·s. The maximum fracture width also varies with viscosity: 0.13 m at 100 mPa·s, 0.14 m at 300 mPa·s, and 0.12 m at 500 mPa·s. This trend indicates that higher viscosity makes it more difficult for the slurry to flow through the coal seam, increasing viscous resistance and reducing fluidity. Consequently, the slurry tends to be retained in fractures. For slurries at 100 and 500 mPa·s, the overall stimulation volume in the later stages of grouting shows a notable decline followed by a rise. In contrast, at 300 mPa·s, there is little fluctuation in late-stage stimulation volume. This behavior results from the dynamic balance between the slurry’s tangential flow and its filtration loss at both ends. Therefore, selecting the appropriate slurry viscosity is crucial for optimizing the stimulation effect.

### 3.2. Influence of Geologic Factors on Diffusion Behavior of Polyurethane Polymer Slurry

**Figure 11:**
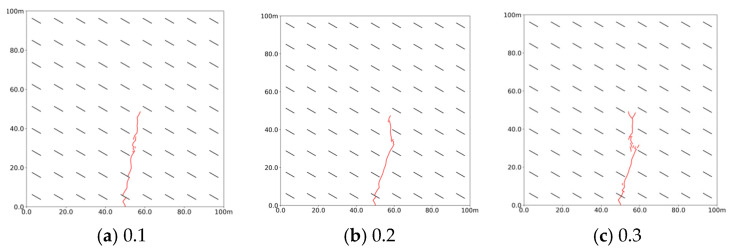
Propagation morphology of porous slurry veins with varying ratios of bedding strength to coal rock strength. (Red lines denote slurry veins) (**a**) 0.1. (**b**) 0.2. (**c**) 0.3.

Figure 11 illustrates that the morphology of slurry veins becomes more complex as bedding strength increases. At a bedding strength to coal rock strength ratio of 0.1, the slurry flows easily along the bedding, expanding a certain distance. However, when this ratio rises to 0.2, the expansion distance decreases. At a ratio of 0.3, the slurry can penetrate the beddings and develop multiple branching veins. This indicates that the influence of natural bedding on the slurry’s expansion pattern only becomes significant when the bedding-to-coal rock strength ratio exceeds a threshold in the range 0.2–0.3. For a ratio ≤ 0.2, veins propagate along bedding; when a ratio ≥ 0.3, they tend to cross beddings and form branches. With the increase in bedding strength, the slurry’s spread length also increases, promoting the formation of multi-branched veins that extend radially and enhance the overall stimulation volume.

**Figure 12:**
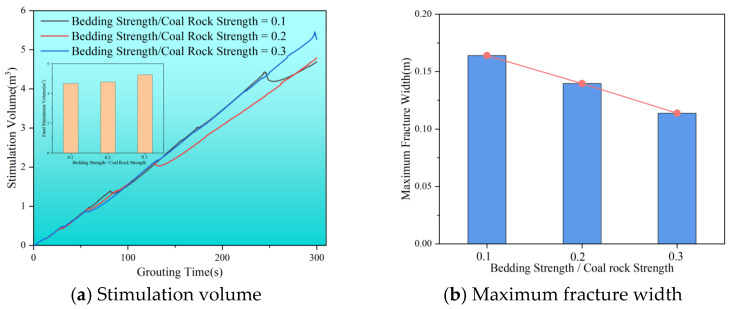
Stimulation volume and maximum fracture width with different ratios of bedding strength to coal rock strength. (**a**) Stimulation volume. (**b**) Maximum fracture width.

Figure 12a illustrates how stimulation volume changes over grouting time for various bedding strength conditions. As the ratio of bedding strength to coal rock strength rises from 0.1 to 0.2, the stimulation range remains relatively constant, suggesting that the ratio of bedding strength to coal rock strength has little effect on stimulation volume. Additionally, the number of fracture closures diminishes with increasing bedding strength. At a bedding strength to coal rock strength ratio of 0.1, the stimulation volume drops significantly twice, by 0.05 m^3^ and 0.23 m^3^. When the ratio increases to 0.2, there is only one notable decline of 0.08 m^3^. At a ratio of 0.3, the stimulation volume becomes nearly proportional to the grouting time, with no fracture closures occurring. This suggests that natural bedding strength plays a crucial role in influencing fracture closure effects. Zhou et al. (2020) [51] found that natural fractures complicate hydraulic fracturing, while Chen et al. (2018) [52] concluded that they increase the number of branching fractures and the stimulation volume. These studies highlight the important role of natural bedding in enhancing fracturing stimulation, consistent with the simulation results in this study. As shown in Figure 12b, the maximum width of the fracture decreases with increasing bedding strength, reaching only 0.11 m at a bedding strength to coal rock strength ratio of 0.3. This indicates that when the bedding strength exceeds a critical ratio relative to the coal matrix (≈0.2–0.3), its influence on the reservoir response increases markedly, promoting splitting rather than localized compaction. This transition enables a larger propagation radius and a greater final stimulation volume. Additionally, Zhu et al. (2009) [53] demonstrated that the fracturing pressure of coal reservoirs significantly decreases in the presence of natural beddings. Thus, when the bedding-to-matrix strength ratio increases (e.g., from ≈0.10 to ≈0.30), its influence on the coal seam’s mechanical response intensifies, making splitting damage more likely than localized compaction.

**Figure 13:**
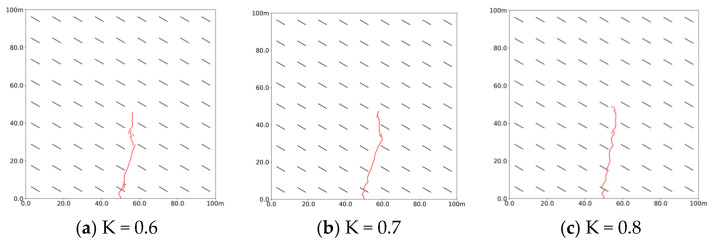
Propagation morphology of porous slurry veins with different lateral pressure coefficients. (Red lines denote slurry veins) (**a**) K = 0.6. (**b**) K = 0.7. (**c**) K = 0.8.

Geostress is a crucial factor influencing the direction and pattern of slurry diffusion. As shown in Figure 13, at a lateral stress coefficient of 0.6, the slurry vein expands along the vertical maximum principal stress without deflecting, but as the coefficient increases to 0.7 and then to 0.9, the slurry begins to flow along the bedding and extends its flow distance due to a diminishing stress difference between vertical and horizontal directions. This trend indicates that a lower lateral pressure coefficient leads to a greater disparity between the maximum and minimum principal stress, with the maximum principal stress exerting the strongest guiding effect on the slurry vein. Consequently, the slurry tends to spread along the vertical direction of the minimum principal stress, as it naturally flows towards areas of lower strength and stress concentration.

**Figure 14:**
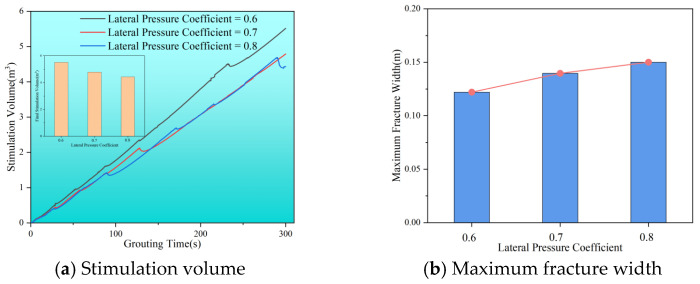
Stimulation volume and maximum fracture width with different lateral pressure coefficients. (**a**) Stimulation volume. (**b**) Maximum fracture width.

The magnitude of ground stress significantly impacts the diffusion radius and characteristics of the slurry vein. As illustrated in Figure 14, increasing the lateral pressure coefficient results in a decrease in stimulation volume while the maximum fracture width gradually increases. Under conditions of high stress difference, the slurry vein tends to exhibit a narrow width and larger volume. Additionally, as the lateral pressure coefficient rises, the stimulation volume continues to decrease, but fracture width expands. This indicates that a smaller lateral pressure coefficient leads to a greater disparity between vertical maximum principal stress and horizontal principal stress, making the reservoir more susceptible to splitting damage. Overall, geostress not only dictate the direction of slurry diffusion but also influence the diffusion radius and the length and width of slurry veins.

### 3.3. Influence of Bedding Characteristics on Diffusion Behavior of Polyurethane Polymer Slurry

**Figure 15:**
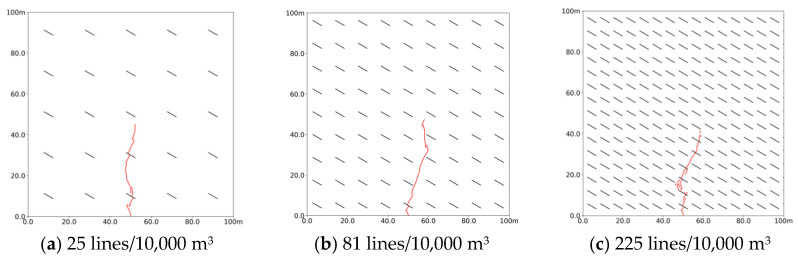
Propagation morphology of porous slurry veins with different bedding densities. (**a**) 25 lines/10,000 m^3^. (**b**) 81 lines/10,000 m^3^. (**c**) 225 lines/10,000 m^3^.

As shown in Figure 15, the morphology of the slurry vein was analyzed at bedding densities of 25, 81, and 225 lines per 10,000 m^3^. At a density of 25 lines/10,000 m^3^, the slurry flows through two bedding layers, deflecting in the direction of bedding inclination and extending over 47 m. At 81 lines/10,000 m^3^, it initially flows through the first bedding layer near the injection port but quickly deviates from the direction of the maximum principal stress, preventing further passage through additional layers. Notably, around 30 m, the slurry steers between the bedding layers, which indicates that natural bedding redistributes stress during directional changes. In this region, the concentration of fluid pressure alters the local maximum principal stress direction. At a bedding density of 225 lines/10,000 m^3^, the slurry shifts from expanding along the bedding to penetrating directly into it, forming fine branching veins and experiencing nearly 90° deflections twice. Guo et al. [54] found that new fractures formed when hydraulic fractures pass through natural beddings always extend in the direction of maximum principal stress. This stress direction is influenced by the reservoir’s initial stress and fluid pressure, meaning that more natural beddings result in more complex stress distribution, affecting fluid flow direction. Additionally, local stress concentration from fluid flow controls reservoir stress redistribution, making the steering effect of natural bedding on hydraulic fractures a result of the interaction between stress and seepage fields.

**Figure 16:**
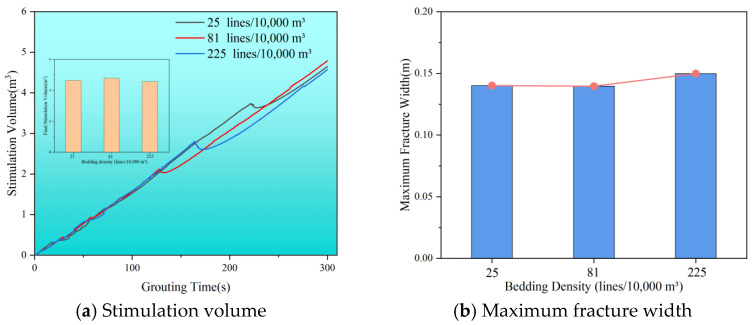
Stimulation volume and maximum fracture width with different bedding surface densities. (**a**) Stimulation volume. (**b**) Maximum fracture width.

Figure 16 shows the stimulation volume change curve during grouting at different bedding densities. The final stimulation volume is approximately the same across all densities, around 4.65 m^3^. However, the timing of fracture closure varies, with the most significant volume reduction occurring at 130 s, 164 s, and 220 s. This variation is linked to how the maximum principal stress responds to bedding density and the timing of reservoir stress redistribution, affecting when fractures experience maximum effective closure pressure. Figure 16b illustrates the maximum width of cracks at different bedding densities. When the bedding density increases from 25 to 81 lines/10,000 m^3^, the maximum fracture width remains nearly constant. This could be due to the limited number of bedding layers the slurry vein can pass through at lower densities of 25 lines/10,000 m^3^, resulting in minimal influence on slurry vein width. Conversely, at higher densities of 225 lines/10,000 m^3^, the more complex stress distribution can lead to greater stress concentration during grouting, increasing crack opening and thickening the slurry vein. Additionally, local stress changes from steering further promote fracture extension, contributing to an increase in vein thickness in those areas.

**Figure 17:**
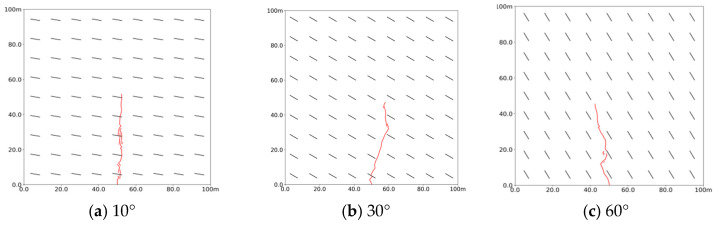
Propagation morphology of porous slurry veins with different bedding angles. (**a**) 10°. (**b**) 30°. (**c**) 60°.

Figure 17 illustrates that the deflection of slurry veins increases significantly with the bedding inclination angle. At a 10° angle, the slurry veins expand almost vertically, with many small branch veins visible. When the bedding inclination is raised to 30°, the slurry veins deflect towards the direction of the maximum principal stress at that angle. At a 60° bedding inclination, the veins become nearly parallel to the bedding direction. This indicates that higher bedding angles lead to more deflected slurry veins, while lower angles result in nearly vertical expansion.

**Figure 18:**
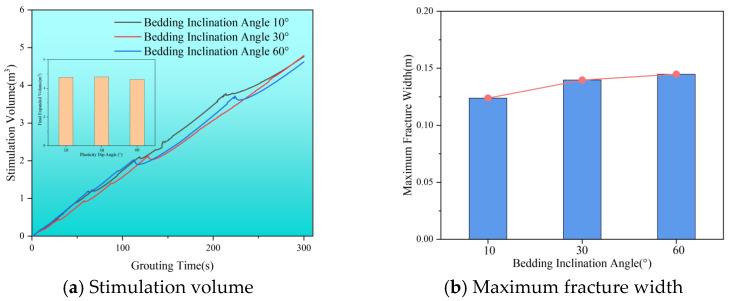
Stimulation volume and fracture width of slurry with different bedding inclinations. (**a**) Stimulation volume. (**b**) Maximum fracture width.

Figure 18a indicates that as bedding inclination angles increase, the stimulation volume remains relatively constant, suggesting that bedding inclination primarily influences the overall morphology of the slurry veins rather than stimulation volume. Figure 18b shows the maximum width of fractures at varying bedding angles. As the angle increases from 10° to 60°, the maximum fracture width grows from 0.12 m to 0.14 m, resulting in noticeably thicker slurry veins. Additionally, the thickness of the slurry vein near the grouting port increases rapidly at the beginning of grouting but slows down later on. The thickness also rises with higher bedding inclination angles. However, the overall stimulation range of the slurry remains consistent, suggesting that the flow path for the slurry is shorter at higher bedding inclinations.

### 3.4. Analysis of Dual-Enhanced Stimulation Process

**Figure 19:**
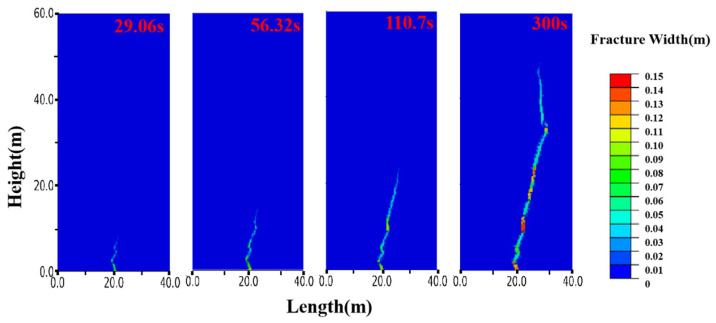
Propagation of Porous Slurry Vein in Coal Seams.

To further investigate the slurry diffusion process, Figure 19 presents the cloud diagram of displacement changes for Run 2. During the first 300 s of injection, the slurry veins experience significant deflection five times, likely due to localized fluid stress concentration at the slurry tip and the diffusion direction toward the maximum principal stress in the reservoir. The color of the slurry vein indicates crack width, with blue representing narrower areas and red indicating thicker regions. This shows that the split-formed slurry vein is nonuniform: it is widest and shortest near the grouting port and narrows and elongates toward the fracture tip, producing a wedge-shaped geometry.

**Figure 20:**
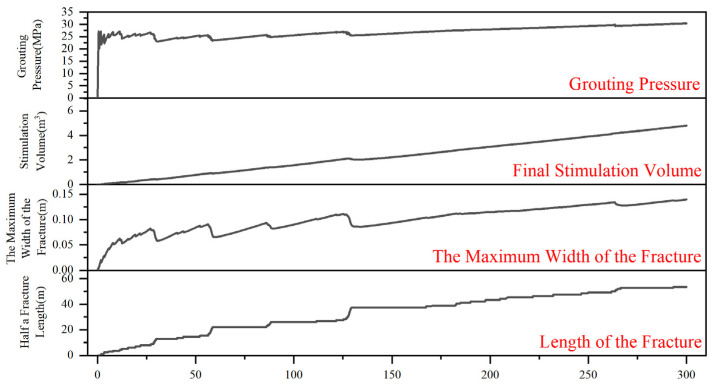
Changes in grouting pressure, stimulation volume, fracture width, and fracture length during the dual-enhanced stimulation process.

As shown in Figure 20, the grouting process comprises three stages: slurry accumulation, splitting grouting, and compaction grouting. In Stage 1, pressure rises rapidly but remains below the fracturing threshold; the slurry accumulates near the injection point, compresses the surrounding coal, and fills pores, elevating pore pressure without creating new fractures. When the grouting pressure approaches ~27 MPa (t ≈ 1–15 s), near-wellbore pores become saturated and the stress at the fracture tip exceeds the damage threshold. Fracture initiation and intermittent propagation then produce pressure oscillations: each advance releases stress, followed by re-pressurization. This pressure cycling characterizes Stage 2 (splitting grouting). A thin, narrow vein forms at the propagating tip, and the first three deflections occur during this phase, consistent with the slurry spreading morphology in Figure 20.

From 15 to 300 s, the grouting pressure stabilizes around 30 MPa, resembling typical fracturing operations. This stability reflects a dynamic balance among leak-off, fracture-volume growth, and injection rate, allowing gradual lateral spreading. Although the pressure remains above the splitting threshold, fractured coal particles accumulate at the slurry front, reducing porosity and raising local fracture resistance. As a result, further fracture extension becomes difficult near ~30 MPa, and compaction grouting becomes dominant. The expansion-volume curve continues to rise while fracture length grows in steps, indicating local accumulation rather than continuous advance. Fluctuations in maximum fracture width suggest partial closure due to the balance between injection and leak-off. Two additional turns occur during the compaction phase, implying uneven in situ stress near the slurry front; these heterogeneities generate local stress concentrations that steer the veins. Each redirection requires additional energy and further impedes propagation.

In conclusion, both splitting and compaction damage occur during dual-enhanced stimulation, with stress concentration at the fracture tips and reservoir stress redistribution being key factors in the slurry vein’s direction.

### 3.5. Analysis of the Main Controlling Factors of Splitting Pressure

**Figure 21:**
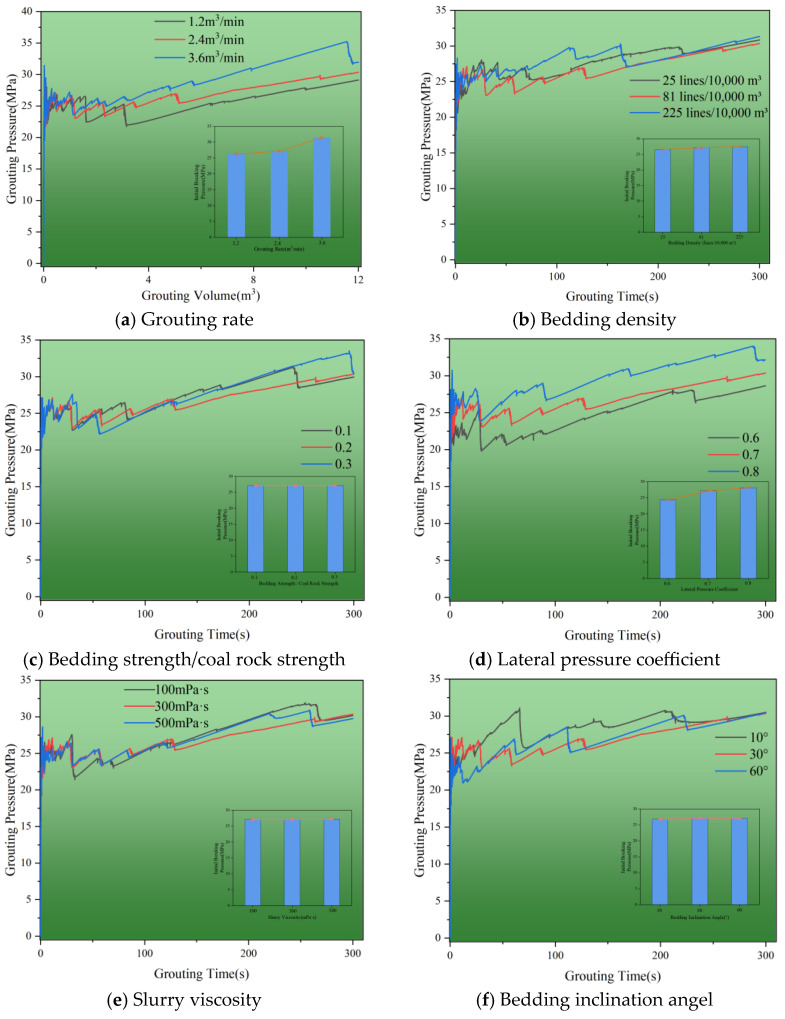
Changes in grouting pressures under different conditions. (**a**) Grouting rate. (**b**) Bedding density. (**c**) Bedding strength/coal rock strength. (**d**) Lateral pressure coefficient. (**e**) Slurry viscosity. (**f**) Bedding inclination angel.

The analysis of grouting pressure under various conditions—such as grouting rates, slurry viscosity, natural bedding strength, lateral pressure coefficient, bedding density, and bedding inclination—aims to identify the primary factors influencing the porous slurry veins propagation. Figure 21 illustrates the grouting pressure curves across different scenarios. Notably, fluctuations in grouting pressure are most prominent during the pre-grouting phase, as the reservoir begins to split and the slurry accumulates in its pore spaces, applying stress to surrounding coal rocks. Once this stress surpasses the fracture strength of the coal rock, a sudden release of pressure occurs, allowing the slurry to propagate forward and create fractures under high fluid pressure. This leads to a repetitive cycle of buildup, release, and filling, establishing seepage channels for slurry expansion. In the mid to late stages of grouting, pressure fluctuations significantly decrease, stabilizing around 30 to 35 MPa, which indicates that the reservoir is experiencing compaction damage. Notably, some runs show a marked reduction in pressure during the late grouting phase, suggesting that the slurry tip pressure has surpassed the fracturing pressure, leading to additional splitting of the reservoir. In this phase, both splitting and compaction grouting modes are present, though compaction damage predominates. The findings reveal that the grouting rate and lateral pressure coefficient are the most influential factors affecting splitting pressure, with variance values of 5.10 and 2.62, respectively. As the grouting rate increases from 1.2 m^3^/min to 3.6 m^3^/min, the initiation pressure rises from 26.28 to 31.44 MPa. This demonstrates that higher grouting rates significantly increase fluid pressure, allowing it to quickly exceed the reservoir’s destructive strength. Consequently, this pressure sensitivity shortens the splitting time, as higher injection rates enable faster filling of the reservoir pores. Therefore, adjusting the grouting rate strategically can effectively reduce splitting pressure while enhancing the diffusion range of the slurry within the reservoir.

## 4. Conclusions

This study presents a novel dual-increasing stimulation method for deep CBM reservoirs. Using the tested mechanical strength and gas permeability of coal rock cores, the finite-discrete element method simulates the propagation of porous slurry veins during dual-increasing stimulation within the CBM reservoir. The research focuses on how engineering parameters, geological factors, and bedding features influence the propagation pattern of slurry veins and the primary factors affecting the fracture initiation pressure in the reservoir. The following conclusions were drawn:

1. As the grouting rate increases, the volume of dual-enhanced stimulation also rises, resulting in a wider maximum width of the slurry vein. Specifically, at a grouting rate of 3.6 m^3^/min, the volume of dual-enhanced stimulation can reach 5.8 m^3^, with the maximum width of the slurry vein measuring approximately 0.15 m. When the slurry viscosity is low, the slurry tends to expand into multiple veins. However, as the viscosity increases, the volume of dual-enhanced stimulation decreases, while the maximum width of the slurry veins expands further. This trend indicates that compaction grouting is more likely to occur at higher viscosities.
2. When bedding strength is high, the slurry tends to penetrate through the beddings. In contrast, a lower bedding strength allows the slurry to expand along the bedding, which decreases the maximum width of the slurry vein as the bedding strength increases. Additionally, as the lateral pressure coefficient rises, the volume of dual-enhanced stimulation decreases, making it more likely for the slurry to expand along the beddings. Natural bedding promotes directional flow; denser bedding results in more pronounced directional changes, creating a more complex network of slurry veins. As the angle of inclination increases, the slurry veins are more likely to change direction. At low bedding angles of 10°, the slurry expands almost vertically, while the maximum width of the slurry vein increases with steeper bedding inclinations.
3. During the grouting process, the directional flow of the slurry may be caused by localized stress concentration at the tip of the slurry, leading to its release along the direction of the maximum principal stress in the reservoir. Pressure fluctuations primarily occur in the early stages of grouting, when the slurry continuously accumulates, releases, and fills within the reservoir, resulting in fracturing damage. In the later stages of grouting, the pressure stabilizes around 30 MPa, with compaction damage becoming predominant. Under these reservoir conditions, both fracturing and compaction grouting modes coexist, with fracturing grouting being more prevalent initially and compaction grouting taking precedence later. Furthermore, the grouting rate significantly affects the fracturing pressure of the reservoir; as the grouting rate increases, the time to achieve fracturing decreases, and the fracturing pressure increases.

## Figures and Tables

**Table 1:** Parameters of numerical simulation model.

Form	Parameters	Value	Data Sources
CBM reservoirs	Modulus of elasticity/GPa	1.7	on-the-spot survey
	Poisson’s ratio	0.25	[41]
	Permeability/mD	1.27	on-the-spot survey
	Failure strength/MPa	9.95	on-the-spot survey
	porosity	0.06	[43]
	Vertical effective geostress/MPa	20	[41,42]
Dual-enhanced slurry	Upper and lower surface loss coefficient/m/(Pa·s)	2.3 × 10^−8^	[46]
	Density/g/cm^3^	1.68	[32]

**Table 2:** Specific parameters in different sets of numerical simulations.

Runs	Grouting Rate (m^3^/min)	Slurry Viscosity (mPa·s)	Lateral Pressure Coefficients	Bedding Strength/Coal Rock Strength	Layer Density (Lines/10,000 m^3^)	Bedding Inclination Angles
1	1.2	300	0.7	0.2	81	30°
2	2.4	300	0.7	0.2	81	30°
3	3.6	300	0.7	0.2	81	30°
4	2.4	100	0.7	0.2	81	30°
5	2.4	300	0.7	0.2	81	30°
6	2.4	500	0.7	0.2	81	30°
7	2.4	300	0.6	0.2	81	30°
8	2.4	300	0.7	0.2	81	30°
9	2.4	300	0.8	0.2	81	30°
10	2.4	300	0.7	0.1	81	30°
11	2.4	300	0.7	0.2	81	30°
12	2.4	300	0.7	0.3	81	30°
13	2.4	300	0.7	0.2	25	30°
14	2.4	300	0.7	0.2	81	30°
15	2.4	300	0.7	0.2	225	30°
16	2.4	300	0.7	0.2	81	10°
17	2.4	300	0.7	0.2	81	30°
18	2.4	300	0.7	0.2	81	60°

## Data Availability

Data is contained within the article.
